# Metformin inhibits intracranial aneurysm formation and progression by regulating vascular smooth muscle cell phenotype switching via the AMPK/ACC pathway

**DOI:** 10.1186/s12974-020-01868-4

**Published:** 2020-06-16

**Authors:** Sichen Li, Yuan Shi, Peixi Liu, Yaying Song, Yingjun Liu, Lingwen Ying, Kai Quan, Guo Yu, Zhiyuan Fan, Wei Zhu

**Affiliations:** 1 Department of Neurosurgery, Huashan Hospital, Fudan University, 12 Wulumuqi Road Middle, Shanghai, 200040 People’s Republic of China; 2 Neurosurgical Institute of Fudan University, Shanghai, People’s Republic of China; 3 Department of Neurology, Renji Hospital, Shanghai Jiao Tong University School of Medicine, No. 160 Pujian Rd, Shanghai, 200025 People’s Republic of China; 4 Neuroscience and Neuroengineering Research Center, Med-X Research Institute and School of Biomedical Engineering, Shanghai Jiao Tong University, Shanghai, People’s Republic of China; 5 Department of Endocrinology and Metabolism, Shanghai Jiao Tong University Affiliated Sixth People’s Hospital, Shanghai, 200233 People’s Republic of China

**Keywords:** Intracranial aneurysm, Metformin, AMPK, Vascular smooth muscle cell, Phenotype modulation

## Abstract

**Background:**

The regulation of vascular smooth muscle cell (VSMC) phenotype plays an important role in intracranial aneurysm (IA) formation and progression. However, the underlying mechanism remains unclear. Metformin is a 5′ AMP-activated protein kinase (AMPK) agonist that has a protective effect on vasculature. The present study investigated whether metformin modulates VSMC phenotype switching via the AMPK/acetyl-CoA carboxylase (ACC) pathway during IA pathogenesis.

**Methods:**

Adult male Sprague-Dawley rats (*n* = 80) were used to establish an elastase-induced IA model. The effects of metformin on AMPK activation and VSMC phenotype modulation were examined. We also established a platelet-derived growth factor (PDGF)-BB-induced VSMC model and analyzed changes in phenotype including proliferation, migration, and apoptosis as well as AMPK/ACC axis activation under different doses of metformin, AMPK antagonist, ACC antagonist, and their combinations.

**Results:**

Metformin decreased the incidence and rupture rate of IA in the rat model and induced a switch in VSMC phenotype from contractile to synthetic through activation of the AMPK/ACC pathway, as evidenced by upregulation of VSMC-specific genes and decreased levels of pro-inflammatory cytokines. AMPK/ACC axis activation inhibited the proliferation, migration, and apoptosis of VSMCs, in which phenotypic switching was induced by PDGF-BB.

**Conclusions:**

Metformin protects against IA formation and rupture by inhibiting VSMC phenotype switching and proliferation, migration, and apoptosis. Thus, metformin has therapeutic potential for the prevention of IA.

## Introduction

Intracranial aneurysm (IA) is a cerebrovascular disease with a global prevalence of up to 7% [1]. Subarachnoid hemorrhage is a consequence of IA rupture that is associated with high morbidity and mortality [2]. The mechanisms underlying IA formation and rupture remain unclear but are thought to be related to pathological changes in vasculature caused by endothelial dysfunction, extracellular matrix remodeling, and phenotype switching of vascular smooth muscle cells (VSMCs) [3–11].

VSMCs are highly specialized contractile cells with principle function to maintain normal vessel morphology and blood pressure [12]. Nowadays, many studies have showed that VSMCs harbor a high degree of plasticity that exhibits dramatic phenotypic changes in response to stimuli [13, 14]. VSMCs could re-differentiate into a phenotype concerned with synthetic activity, deemed phenotypic switching. This process involves coordinated downregulation of contractile proteins such as α-smooth muscle actin (α-SMA) and smooth muscle protein 22α (SM-22α) and increased production of factors that mediate inflammation such as matrix metalloproteinases (MMPs) and tumor necrosis factor-α (TNF-α), among others [15, 16]. The pro-inflammatory role of VSMCs synthetic phenotype may result in extracellular remodeling and the destruction of the media, leading to the formation and rupture of IA [6–10]. AMP-activated protein kinase (AMPK) is a highly conserved protein kinase that plays a critical role in cell and organ metabolism. It was recently suggested that AMPK has a vasculoprotective effect [16, 17]. Acetyl-coenzyme A carboxylase (ACC) is a downstream effector of AMPK that is involved in the regulation of fatty acid metabolism [18, 19]. ACC regulates endothelial cell migration and has been implicated in vascular diseases [20]. Metformin, an AMPK activator, exerts a cardioprotective function by reducing the incidence of cardiovascular events [21]. However, it is unclear whether it provides protection against IA. In the present study, we tested the hypothesis that metformin inhibits IA development and progression by modulating VSMC phenotype and function and investigated the underlying mechanism.

## Methods

### Patient samples

Ten IA samples and 5 normal superficial temporal artery (STA) tissue, which served as a control, were obtained from 15 patients undergoing surgical IA clipping. The samples were fixed in 10% formaldehyde and embedded in paraffin. Five-micrometer sections were placed on polylysine-coated slides prior to HE staining. The use of human tissues was approved by the ethics committee of Huashan Hospital, Fudan University, and followed the 1964 Declaration of Helsinki. Written informed consent was obtained from each patient.

### Rat IA model

Animal experiments were carried out according to a protocol approved by the Institutional Animal Care and Use Committee of Huashan Hospital, Fudan University. Male Sprague-Dawley rats (6–8 weeks old and weighing 180–200 g; Shanghai JiesiJie Experimental Animal Co., Shanghai, China) were randomized using a computer-generated algorithm into four groups (*n* = 20 each) that were treated with 100 mg/kg metformin (PHR1084), 20 mg/kg compound C (P5499) (both from Sigma-Aldrich, St. Louis, MO, USA), or their combination. The compounds were separately dissolved in normal saline and 20% dimethyl sulfoxide and intraperitoneally injected into the rats once daily. Rats in the control group were injected with 20% dimethyl sulfoxide.

A rat IA model was induced by elastase as previously described [22, 23]. Briefly, rats were placed in the supine position and anesthetized by inhalation of 3% isoflurane. The right common carotid artery was ligated with a 4-0 nylon thread. After drilling a small burr hole, 10 μl elastase (E1250, Sigma-Aldrich, St. Louis, MO, USA) was injected into the right basal cisterns based on stereotactic coordinates. Hypertension was induced by feeding the rats a high-salt diet for 30 days. Baseline measurements of systolic arterial blood pressure (SBP), blood glucose (BG), and serum sodium (Na^+^) and potassium (K^+^) concentrations were recorded before and 0, 1, 2, 3, and 4 weeks after IA induction. After 30 days, the rats were euthanized by CO_2_ overdose and perfused with phosphate-buffered saline (PBS) and 4% paraformaldehyde (PFA), then infused with 2% India ink or plastic using Batson’s No. 17 plastic kit (07349; Polysciences, Warrington, PA, USA). The brain samples were processed as described above for human samples.

Aneurysm formation was defined as artery dilation to a diameter greater than 50% of that of the parent artery and was determined through microscopic observation by two independent observers who were blinded to group assignment. Aneurysm formation was graded as follows: grade 1, normal artery; grade 2, aneurysmal dilation without aneurysm formation; grade 3, unruptured aneurysm(s); and grade 4, ruptured aneurysm(s).

### Hematoxylin and eosin (HE) and Masson’s trichrome staining

Tissue samples were sectioned at a thickness of 5 μm. The sections were collected on polylysine-coated slides and stained with the HE Stain Kit (HT25A-1KT) and Trichrome Stain Kit (HT-15KT) (both from Sigma-Aldrich) according to the manufacturer’s protocol.

### Scanning electron microscopy

After transcardial perfusion with Batson’s No. 17 plastic kit, cerebral vascular corrosion casts were prepared as previously described [24]. Briefly, whole brain tissue was digested with 20% KOH for 24 h at room temperature; excess tissue was removed by intermittently rinsing with water. The surface of the vascular cast was sprayed with colloidal silver paste and the sample was examined with a scanning electron microscope (SU8010; Hitachi, Tokyo, Japan).

### Immunofluorescence analysis

Rat brain tissue sections and fixed VSMC cells were incubated overnight at 4 °C with antibodies against α-SMA (ab7817, 1:200 dilution), SM22α (ab10135, 1:250 dilution), and phosphorylated AMPK (p-AMPK) (ab23875, 1:150 dilution) (all from Abcam, Cambridge, UK), followed by the appropriate fluorophore-labeled secondary antibody(A11055, A21203, and A21206, 1:1000 dilution, all from Thermo Fisher Scientific, MA, USA). The nuclei were counterstained with 4′,6-diamidino-2-phenylindole (C1002; Beyotime Institute of Biotechnology, Shanghai, China), and the sections were imaged with a laser scanning confocal microscope (Leica Microsystems, Wetzlar, Germany). For each analysis, at least three sections/wells were selected, and five visual fields were randomly observed from each section/well with 100 cells from each field, with the percentage of positive cells calculated accordingly.

### Primary VSMC isolation and culture

Rat VSMCs were isolated and cultured as previously described [25]. Briefly, the cells were isolated from the aorta and cultured in Dulbecco’s minimum essential medium (DMEM; Gibco, Grand Island, NY, USA) containing 10% fetal bovine serum (FBS; Gemini, West Sacramento, CA, USA), 100 U/ml penicillin, and 100 mg/ml streptomycin at 37 °C and 5% CO_2_. VSMCs were identified based on positive anti-α-SMA antibody labeling (1:300) and cell morphology. Cells between passages 3 and 8 were used in experiments.

### Induction of VSMC phenotype switching

VSMCs were seeded in a 6-well plate at a density of 1 × 10^6^ cells/ml in DMEM containing 10% FBS and incubated at 37 °C and 5% CO_2_ for 24 h. The medium was replaced with serum-free DMEM containing platelet-derived growth factor (PDGF)-BB (Peprotech, Rocky Hill, NJ, USA) at 10 ng/ml and incubated at 37 °C for 24 h to stimulate phenotype switching [26].

### VSMC treatment and experimental design

When they reached confluence, VSMCs were incubated in DMEM containing 10% FBS and different concentrations of metformin (5, 50, and 500 μM), 10 μM compound C, or 500 nM ND-646 (ACC inhibitor; MedChemExpress, Monmouth Junction, NJ, USA), or a combination of metformin and ND-646. After 24 h, VSMCs were harvested for analysis.

### Cell proliferation and migration assays

The proliferation of VSMCs was evaluated with Cell Counting Kit (CCK)-8 (Dojindo Molecular Technologies, Rockville, MD, USA) according to the manufacturer’s protocol. Briefly, VSMCs were seeded in a 96-well plate at 5 × 10^3^ cells/well in triplicate and treated with different doses of metformin and inhibitor for 24 h. A 10-μl volume of CCK-8 solution was added to each well and the cells were cultured in a humidified atmosphere of 95% air and 5% CO_2_ at 37 °C for various times. The absorbance at 450 nm was measured with a microplate reader (Bio-Tek, Winooski, VT, USA).

Cell migration was assessed with transwell permeable supports (24-well, 3.0-μm membrane; Corning Inc., Corning, NY, USA). VSMCs (5 × 10^4^ cells/ml) were seeded in the upper chamber with serum-free DMEM and treated with metformin and inhibitors; DMEM with 10% FBS was added to the lower chamber. After incubation at 37 °C and 5% CO_2_ for 24 h, cells that had migrated into the upper chamber were fixed with 4% PFA, stained with crystal violet solution (Beyotime Institute of Biotechnology), and counted under a light microscope.

### Hoechst staining

VSMCs were seeded in 24-well plates at 10^4^ cells/well and cultured at 37 °C in humidified 95% air/5% CO_2_ until they reached 60% confluence. Following treatment, the cells were fixed and washed three times with PBS, and 0.2 ml Hoechst (Beyotime Institute of Biotechnology) was added to each well for 5 min. The samples were washed three times and visualized under a laser scanning confocal microscope. Cells were quantified using ImageJ v1.52q software (National Institutes of Health, Bethesda, MD, USA).

### Quantitative real-time (qRT)-PCR

Total RNA was extracted from VSMCs using TRIzol reagent (Takara Bio, Otsu, Japan) according to the manufacturer’s protocol. ABScript II cDNA First-Strand Synthesis Kit (Abclonal Technology, Woburn, MA, USA) was used to synthesize cDNA, and qRT-PCR was performed using a One-Step SYBR Prime Script RT-PCR Kit II (Takara Bio) by Real-Time PCR system (ABI 7500, Thermo Fisher Scientific, MA, USA). Glyceraldehyde 3-phosphate dehydrogenase (GAPDH) was used as the internal control, and relative mRNA levels were determined using the 2^−ΔΔCt^ method.

### Western blotting

Cells were lysed radioimmunoprecipitation assay buffer (Beyotime Institute of Biotechnology) and proteins were separated by sodium dodecyl sulfate polyacrylamide gel electrophoresis and transferred to a polyvinylidene difluoride membrane (Bio-Rad, Hercules, CA, USA) that was blocked with 5% fat-free dry milk in Tris-buffered saline containing 0.1% Tween 20 (TBST) for 1 h, then incubated overnight at 4 °C with primary antibodies against α-SMA (1:200), SM22α (1:250), AMPK (1:200), p-AMPK (1:200), ACC (1:250), p-ACC (1:250), and GAPDH (1:1000) (all from Abcam). The membrane was then incubated with horseradish peroxidase-conjugated IgG for 1 h at room temperature, and protein bands were visualized with a chemiluminescence kit (Beyotime Institute of Biotechnology) and X-ray imaging system (Tanon, Shanghai, China). Signal intensity was quantified with ImageJ software.

### Statistical analysis

Experiments were performed at least three times. Data are expressed as mean ± standard deviation and were analyzed with SPSS v23.0 software (IBM, Armonk, NY, USA). The statistical significance of differences between groups was evaluated by one-way analysis of variance, with *P* < 0.05 considered significant.

## Results

### VSMCs in human IA samples switch from a contractile to synthetic phenotype with the downregulation of p-AMPK

**Fig. 1:**
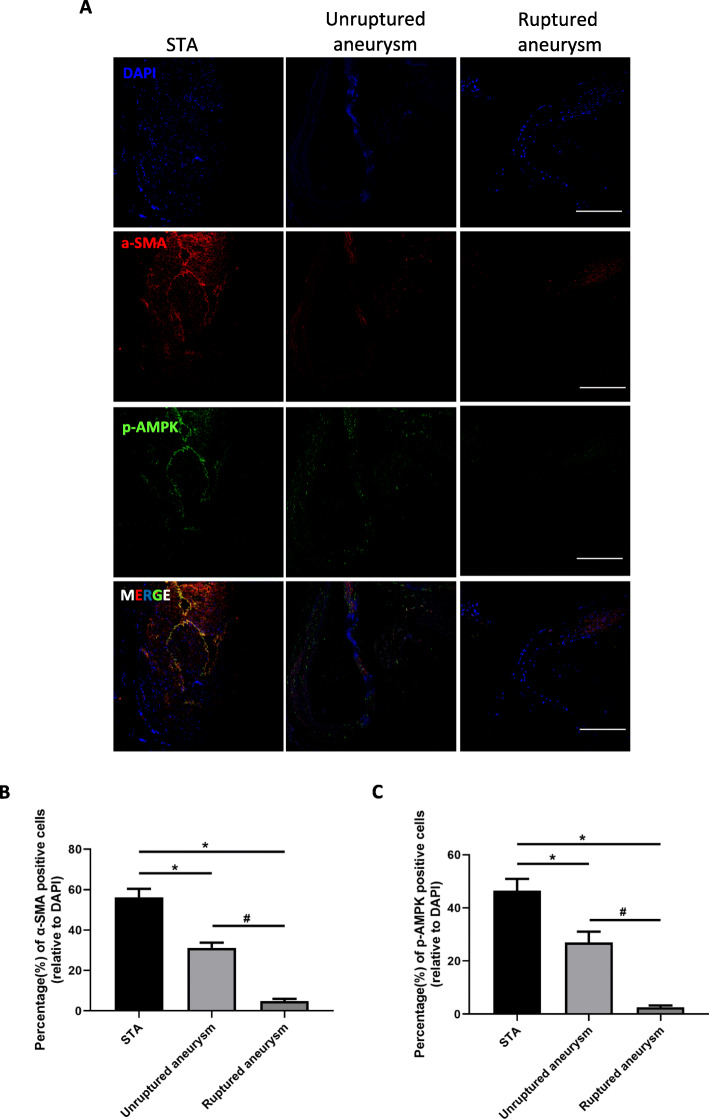
Decreased expression of p-AMPK and αSMA in human unruptured and ruptured IA tissue compared to STA. STA and IA were double-labeled with antibodies against α-SMA (red) and p-AMPK (green) and stained with 4′,6-diamidino-2-phenylindole (DAPI; blue). Compared to STA, p-AMPK and α-SMA expression was lower in unruptured and ruptured IA vessel walls. **a** Representative immunofluorescence images of human tissues. **b**, **c** Quantitative analysis of cells positive for α-SMA (**b**) and p-AMPK (**c**). *P < 0.05 vs STA. ^#^*P* < 0.05 vs ruptured IA. Scale bar = 20 μM

To investigate the relationship between AMPK activation and phenotype modulation in IA, we evaluated α-SMA and p-AMPK expression levels in human IA and STA tissues. In the latter, α-SMA and p-AMPK were expressed at high levels and the cells had a normal morphology (Fig. 1). In contrast, in the IA group, both markers were downregulated in the medial and adventitial layers. α-SMA and p-AMPK levels were significantly lower in ruptured as compared to unruptured IA tissue (α-SMA-positive cells, 4.88% vs 31.28%, *P* < 0.05; p-AMPK positive cells, 2.64% vs 27.04%, *P* < 0.05.), or STA tissue (α-SMA-positive cells, 4.88% vs 56.2%, *P* < 0.05; p-AMPK positive cells, 2.64% vs 46.56%, *P* < 0.05). These findings suggest that VSMCs undergo phenotype switching in IA, which is accompanied by decreased expression of p-AMPK.

### Metformin prevents IA formation and progression in a rat model

**Fig. 2:**
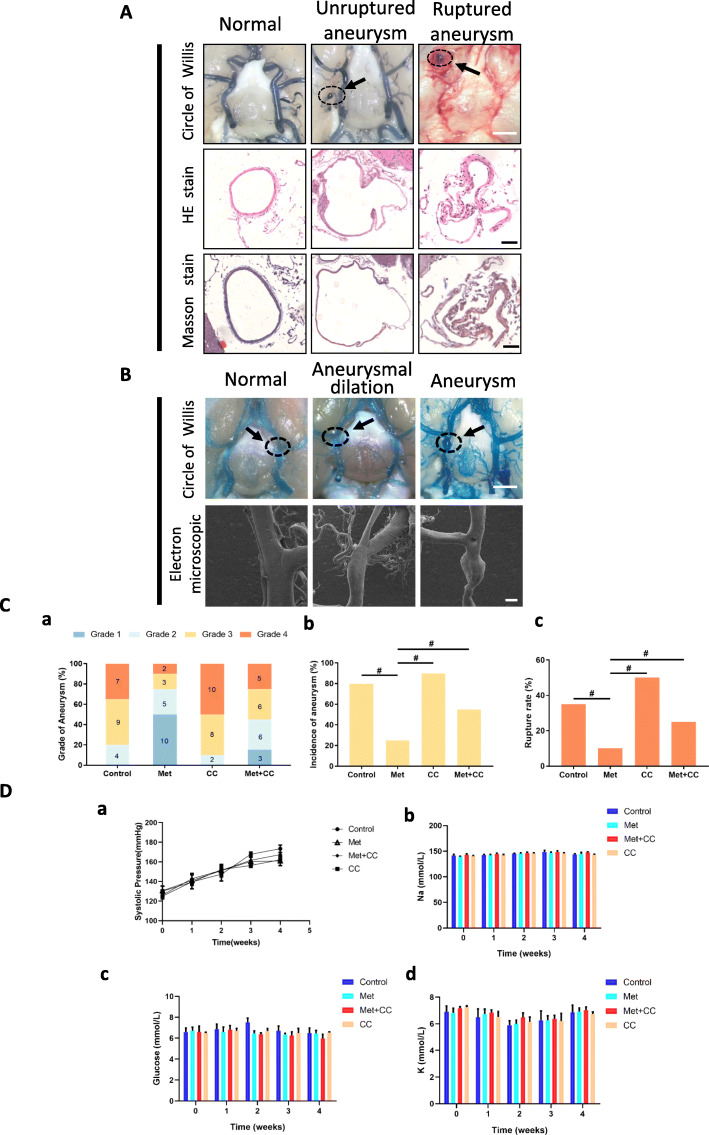
Metformin suppresses IA formation and progression in a rat model. **A** Representative images of cerebral arteries. HE and Masson’s trichrome staining show the destruction of vessel walls (arrow) in a rat model of IA. Scale bar = 50 μM. **B** Scanning electron micrographs of cerebral vessels; high magnification views reveal local bulging of vessels. Scale bar = 200 μM. **C** (a) Quantitative analysis of IAs in control, metformin, compound C, and metformin + compound C groups (*n* = 20/group). (b, c) Rate of aneurysm formation (b) and rupture (c) in different treatment groups (*n* = 20/group). ^#^*P* < 0.05 vs 500 μM metformin group. (**D**) Changes in physiological parameters at indicated time points after IA induction. (a) Systolic pressure. (b, d) Serum Na^+^ (b) and K^+^ (d). (c) Blood glucose

We examined the effect of metformin on VSMC phenotype switching during the formation and progression of IA in a rat model. Local bulging was observed in the circle of Willis in IA rats by microscopy (Fig. 2A). To better visualize the ruptured aneurysm, most of the blood clots in the subarachnoid space were removed. HE staining revealed a thinning of the vessel wall in both unruptured and ruptured IA, and Masson’s trichrome staining showed severe degradation of the tunica media compared to normal vessels (Fig. 2A). Electron microscopy analysis of normal vessels, aneurysmal dilation without IA formation, and unruptured IA (Fig. 2B) revealed different grades of aneurysm in the four groups (Fig. 2C (a)). IA incidence was lower in the metformin group than in control samples (25% vs 80%, *n* = 20; *P* < 0.05) (Fig. 2C (b)), samples treated with the p-AMPK antagonist compound C (25% vs 90%, *n* = 20; *P* < 0.05) (Fig. 2C (b)), or both metformin and compound C (25% vs 55%, *n* = 20; *P* < 0.05) (Fig. 2C (b)). Treatment with p-AMPK agonist also had a protective effect against IA rupture relative to the control group (10% vs 35%; *P* < 0.05) (Fig. 2C (c)) and compound C group (10% vs 50%; *P* < 0.05) (Fig. 2C (c)).

To confirm the protective effect of metformin against IA formation and progression, compound C was added to the metformin-treated samples. We found that both the incidence of IA and rupture rate were normalized by adding compound C compared to metformin treatment alone (incidence, 25% vs 55% and rupture rate, 10% vs 25%; both *P* < 0.05, *n* = 20) (Fig. 2C (b), (c)). No differences were observed between experimental and control groups in terms of SBP, BG, and Na^+^ and K^+^ concentrations (Fig. 2D).

### Metformin inhibits VSMC phenotype switching via AMPK activation in IAs

**Fig. 3:**
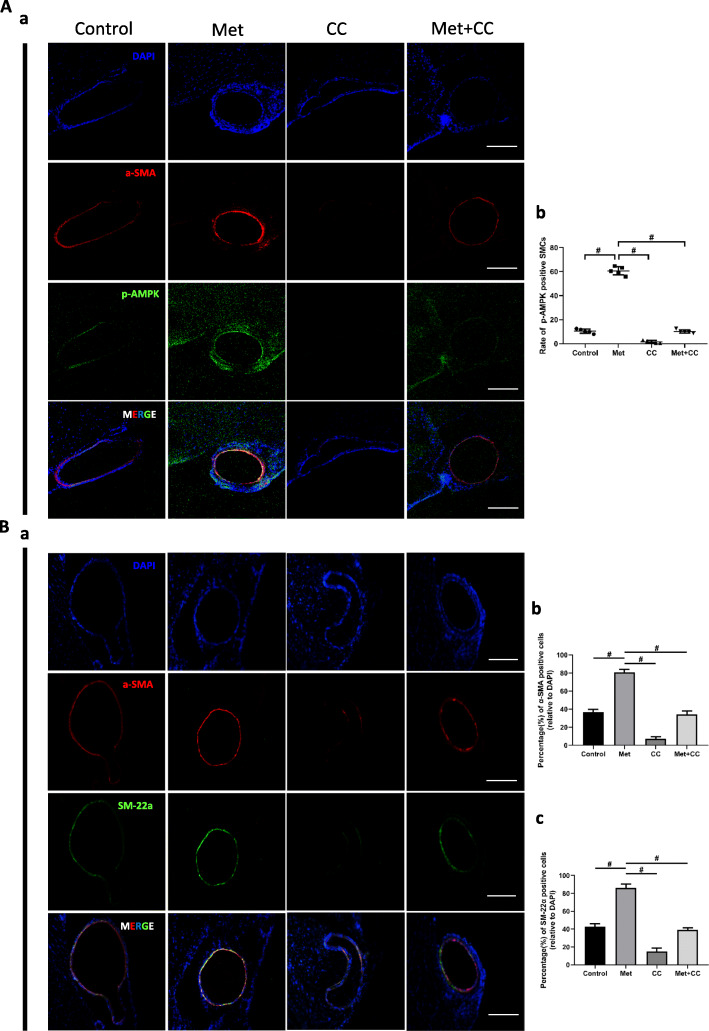
Metformin inhibits p-AMPK and contractile marker expression in VSMCs in a rat IA model. **A** Representative images and quantitative analysis of α-SMA (red) and p-AMPK (green) expression in rat IAs in control, metformin, compound C, and metformin+ compound C groups; nuclei were counterstained with 4′,6-diamidino-2-phenylindole DAPI (blue). Metformin increased the expression of α-SMA and p-AMPK. (a) Representative images of immunofluorescence in rat IA sections. (b) Quantitative analysis of p-AMPK-positive VSMCs. **B** Representative images and quantitative analysis of α-SMA (red) and SM22α (green) immunofluorescence in VSMCs. Metformin enhanced the expression of both markers. (a) Representative images of immunofluorescence labeling of rat IA sections. (b, c) Quantitative analysis of α-SMA-positive (b) and SM22α-positive (c) cells. ^#^*P* < 0.05 vs 500 μM metformin group. Scale bar = 50 μM

To investigate the mechanism by which metformin prevents IA formation and progression, we examined α-SMA, SM22α, and p-AMPK expression in rat IAs by immunofluorescence labeling. Compared to the control group, metformin treatment increased the levels of p-AMPK (Fig. 3A) and α-SMA and SM22α (Fig. 3B), providing evidence for VSMC phenotype switching. Compound C reversed this effect (Fig. 3A, B); however, the decrease in expression was rescued by combined application of compound C and metformin (Fig. 2C).

### AMPK phosphorylation stimulates VSMC contractility-related gene expression and suppresses inflammatory mediators

**Fig. 4:**
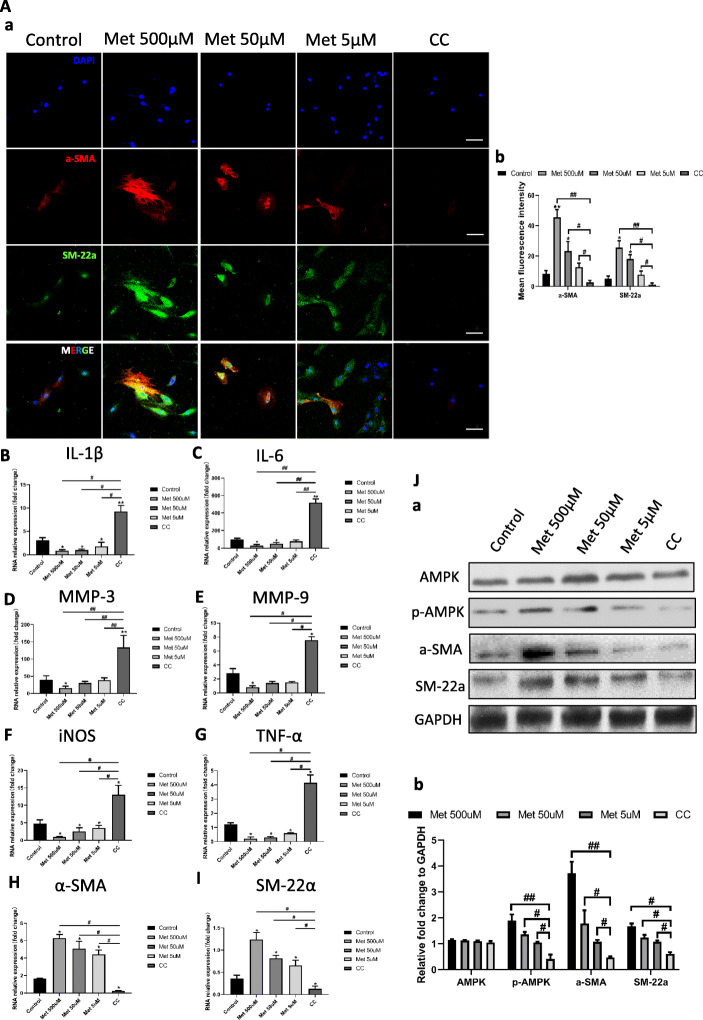
AMPK phosphorylation stimulates contractility-related gene expression and decreases the levels of inflammatory mediators in PDGF-BB-induced VSMCs. **A** Representative images and quantitative analysis of α-SMA (red) and SM22α (green) expression in PDGF-BB-induced VSMCs treated with 5, 50, or 500 μM metformin and compound C. (a, b) Representative images of α-SMA and SM22α immunofluorescence in PDGF-BB-induced VSMCs (a) and quantitative analysis of mean fluorescence intensity (b). **B**–**G** qRT-PCR analysis of IL-1β, IL-6, MMP-3, MMP-9, iNOS, and TNF-α expression in PDGF-BB-induced VSMCs treated as indicated. **H**, **I** qRT-PCR analysis of α-SMA and SM22α levels. **J** Western blot analysis of total AMPK, p-AMPK, α-SMA, SM22α, and GAPDH in VSMCs treated as indicated. (a, b) Representative immunoblot (a) and quantification (b).*P < 0.05, **P < 0.01 vs control group; ^#^*P* < 0.05, ^##^*P* < 0.01 vs 500 μM metformin group. Scale bar = 50 μM

To further assess the effect of metformin on VSMC phenotype switching, PDGF-BB-induced VSMCs (P-VSMCs) were used to simulate VSMCs in the IA vessel wall as previously described [26]. AMPK phosphorylation level was enhanced by metformin in a dose-dependent manner (Fig. 4A, J). This was accompanied by upregulation of α-SMA and SM22α relative to the control group (Fig. 4A, H–J). The most potent activation was observed at the highest concentration of metformin that we tested (500 μM). Treatment with the AMPK inhibitor compound C at 10 μM decreased AMPK phosphorylation along with α-SMA and SM22α expression (Fig. 4A, H–J).

Given that downregulation of SMC contractility-related genes and increased production of inflammatory mediators are markers of VSMC phenotype switching, we evaluated the expression of inflammation-related genes by qRT-PCR. The levels of the pro-inflammatory factors interleukin (IL)-1β, IL-6, MMP-3, MMP-9, inducible nitric oxide synthase (iNOS), and TNF-α were decreased by treatment with metformin relative to the control group (Fig. 4B–G) but were increased in the compound C group, which was associated with the downregulation of α-SMA and SM22α (Fig. 4H, I). These results indicate that metformin inhibits VSMC phenotype switching by inducing AMPK phosphorylation.

### Metformin inhibits VSMC proliferation, migration, and apoptosis

**Fig. 5:**
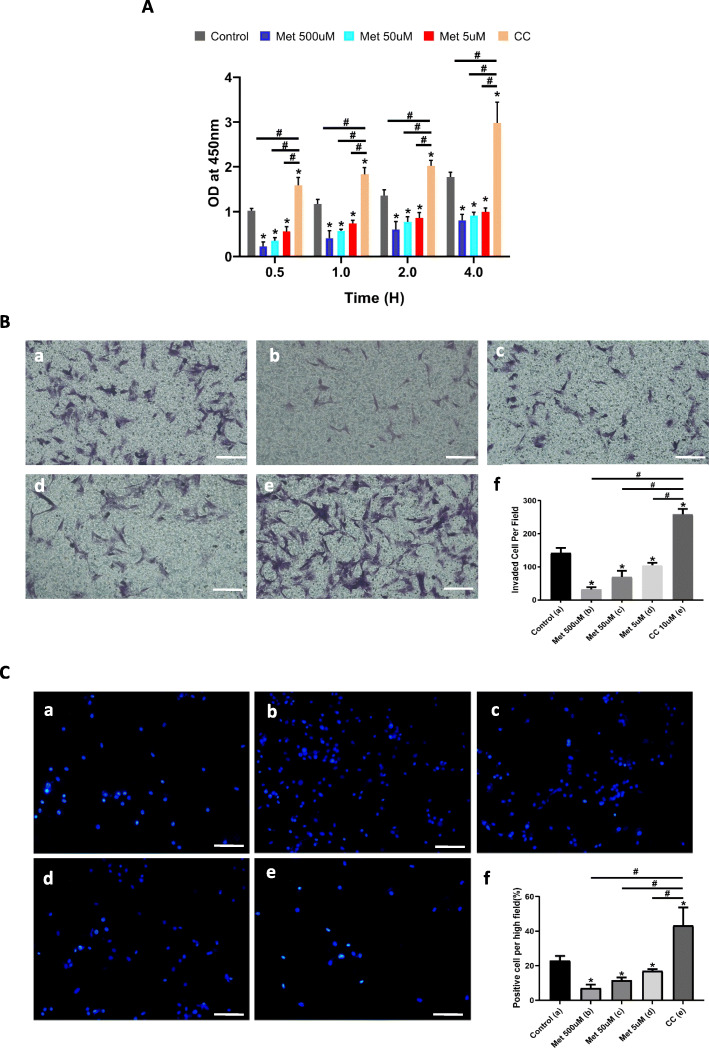
Metformin inhibits VSMC proliferation, migration, and apoptosis via AMPK activation. **A** Cell proliferation assay measured with CCK-8 at 0.5, 1, 2, and 4 h after incubated with 5, 50, and 500 μM metformin and compound C. **B** Evaluation of migratory capacity with the transwell assay. (a–e) Representative images of migrating cells stained with crystal violet are shown for the control (a), 500 μM metformin (b), 50 μM metformin (c), 5 μM metformin (d), and 10 μM compound C (e) groups. Scale bar = 50 μM. **C** (a–e) Representative images and quantitative analysis of apoptotic cells analyzed by Hoechst staining in the control (a), 500 μM metformin (b), 50 μM metformin (c), 5 μM metformin (d), and 10 μM compound C (e) groups. Scale bar = 50 μM. (f) Quantitative analysis. *P < 0.05 vs control group; ^#^*P* < 0.05 vs 500 μM metformin group. OD, optical density

Cellular activities such as proliferation and migration are inactive in contractile VSMCs [9, 15]. To investigate the effect of metformin on these activities, we evaluated cell proliferation with the CCK-8 assay and found that metformin suppressed the proliferative capacity of VSMCs in a dose- and time-dependent manner; compound C had the opposite effect, with significant differences observed between control, metformin, and compound C groups (Fig. 5A). We also assessed VSMC motility with the transwell assay and found that metformin inhibited VSMC migration (Fig. 5B (b)–(d), (f)), which was stimulated by compound C relative to the control and metformin groups (Fig. 5B (a), (e), (f)).

After phenotype switching during aneurysm formation, VSMCs may undergo apoptosis, ultimately resulting in aneurysm rupture [27]. We examined VSMC apoptosis by Hoechst staining and observed that it was decreased by metformin treatment (Fig. 5C (b)–(d), (f)) and increased by compound C via downregulation of p-AMPK (Fig. 5C (e), (f)).

### AMPK/ACC signaling contributes to VSMC phenotype switching

**Fig. 6:**
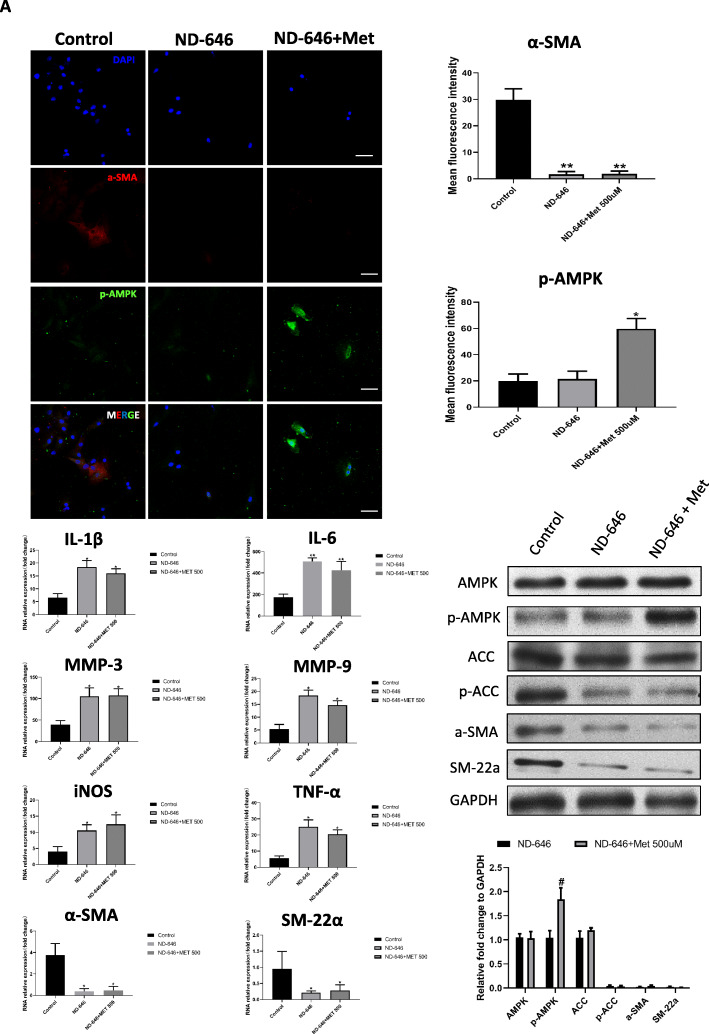
Metformin modulates VSMC switching phenotype via AMPK/ACC signaling. **A** Representative images and quantitative analysis of α-SMA (red) and p-AMPK (green) expression in control, ND-646, and ND-646 + metformin groups following PDGF-BB induction. (a) Representative immunofluorescence images of PDGF-BB-induced VSMCs. (b, c) Quantitative analysis of mean fluorescence intensity of α-SMA (b) and p-AMPK (c). **B**–**G** qRT-PCR analysis of IL-1β, IL-6, MMP-3, MMP-9, iNOS, and TNF-α expression in PDGF-BB-induced VSMCs treated as indicated. **H**, **I** qRT-PCR analysis of α-SMA and SM22α mRNA levels. **J** Western blot analysis of total AMPK, p-AMPK, ACC, p-ACC, α-SMA, SM22α, and GAPDH expression in VSMCs treated as indicated. (a, b) Representative western blot (a) and quantification (b).*P < 0.05, **P < 0.01 vs control group; ^#^*P* < 0.05 vs ND-646 group. Scale bar = 50 μM

ACC is a downstream effector of AMPK that plays a critical role in fatty acid metabolism [18, 19] and has been implicated in vascular diseases [20]. To clarify the molecular basis for VSMC phenotype switching, we used ND-646, a small molecular inhibitor of ACC [28]. The level of p-ACC was decreased by exposure to ND-646, with no observable difference in p-AMPK expression (Fig. 6A, J). A qRT-PCR analysis revealed that α-SMA and SM22α were downregulated in the presence of ND-646 (Fig. 6H, I), which was corroborated by immunofluorescence labeling (Fig. 6A) and western blotting (Fig. 6J). In contrast, expression of the inflammatory mediators IL-1β, IL-6, MMP-3, MMP-9, iNOS, and TNF-α was increased in the ND-646 group (Fig. 6B–G). Treatment with 500 μM metformin enhanced p-AMPK expression (Fig. 6A, J) while suppressing that of the contractility markers α-SMA and SM22α (Fig. 6A, H–J). There was no difference in the levels of inflammation-related factors between the ND-646 and ND-646 + metformin groups (Fig. 6B–G). These results indicate that metformin modulates VSMC phenotype switching via the AMPK/ACC pathway.

### VSMC proliferation, migration, and apoptosis are regulated by the AMPK/ACC signaling pathway

**Fig. 7:**
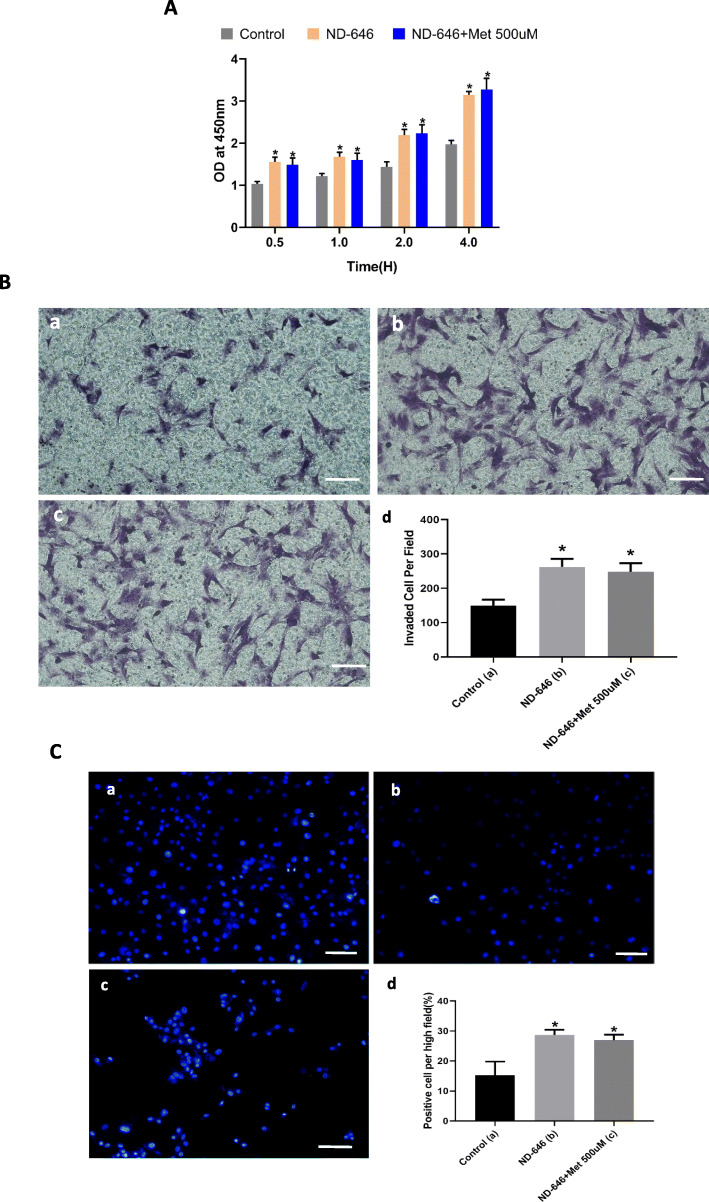
VSMC proliferation, migration, and apoptosis are regulated via AMPK/ACC signaling. **A** Cell viability following treatment with ND-646 and ND-646 + metformin at indicated times. **B** Evaluation of VSMC migration with the transwell assay. Scale bar = 50 μM). (a–d) Representative images of control (a), (b) ND-646 (b), and ND-646 + 500 μM metformin (c) groups and quantitative analysis (d). **C** Apoptotic cells detected by Hoechst staining. Scale bar = 50 μM. (a–d) Representative images of control (a), (b) ND-646 (b), and ND-646 + 500 μM metformin (c) groups and quantitative analysis (d). *P < 0.05 vs control group. OD, optical density

The results of CCK-8 assay indicated that the proliferative capacity of VSMCs was enhanced by treatment with ND-646 (Fig. 7A), which was not abrogated in the presence of metformin (Fig. 7A). Consistent with these findings, VSMC migration was stimulated by ND-646 (Fig. 7B (b), (d)) and that mobility was not restored by adding metformin (Fig. 7B (c), (d)). ND-646 treatment increased apoptosis in VSMCs (Fig. 7C (b), (d)) relative to stimulation with metformin. ND-646 + metformin had similar effects on apoptosis, proliferation, and migration (Fig. 7C (c), (d)).

## Discussion

The pathogenesis of IA is not fully understood and there are no drugs that have been shown to be clinically effective in preventing IA formation, progression, and rupture. In a feasibility study, minocycline and doxycycline had a stabilizing effect and prevented the rupture of IAs in a mouse model [29]. Membrane-associated prostaglandin E synthase (mPEGS)-1 plays a protective role in blood vessels, and aspirin was shown to attenuate cerebral aneurysm rupture and reduce mortality in a mouse mPGES-1 deficiency model of IA [30]. Another glucose-regulating drug, pioglitazone, prevented aneurysm rupture through activation of peroxisome proliferator-activated receptor γ in mouse macrophages [31]. Despite the therapeutic potential of these compounds, there are no drugs that are known to suppress aneurysm formation. Metformin is a widely used glucose-lowering drug that has multiple clinical benefits including anti-tumor effects, attenuation of autoimmune disorder, and cardiovascular protection [16, 17, 32, 33]. Although the underlying mechanisms are not fully understood, metformin is thought to act via activation of AMPK [34, 35]. In our study, administration of metformin in rats decreased the incidence and rupture rate of IAs, with concomitant upregulation of p-AMPK, α-SMA, and SM22α. Meanwhile, treatment with the AMPK antagonist compound C had the opposite effect. These results indicate that metformin inhibits IA formation and progression via an AMPK-dependent pathway, in agreement with the findings from our analysis of human IA and STA samples.

Many studies on the etiology of IAs have focused on hemodynamic changes, aneurysm-intrinsic biological factors, inflammation, and vascular remodeling [6, 7, 27]. VSMCs are an important cell type in the vascular tunica media for their role in maintaining the integrity of cerebral vasculature. In contrast to extracranial arteries, the tunica media constitutes the largest part of the cerebral artery wall whereas the tunica adventitia and elastic fibers are sparse [36]. In the case of injury to the endothelium, VSMCs switch their phenotype from contractile to pro-inflammatory and pro-matrix (i.e., synthetic) [12, 37]; these changes in the differentiation potential and synthetic capacity of VSMCs, which are accompanied by increased production of inflammatory mediators and MMPs, contribute to IA rupture [27, 38]. After phenotype switching, VSMCs lose the phenotype and undergo apoptosis, which is the final step in the cascade of events leading to IA rupture [27]. Clarifying the pathogenesis of VSMC phenotype modulation is therefore critical for understanding the mechanism of IA formation and progression.

The mechanistic basis for phenotype switching by VSMCs is complex and has not been fully elucidated. Endogenous reactive oxygen species were shown to promote the maturation and differentiation of VSMCs via p38 mitogen-activated protein kinase signaling [39], while activation of the phosphatidylinositol 3-kinase/protein kinase B pathway was determined to play an important role in Notch-mediated VSMC phenotype conversion [40]. Moreover, the cyclic AMP kinase/protein kinase A/cAMP response element-binding protein signaling axis also regulates phenotypic changes in VSMCs by mediating cilostazol-induced VSMC differentiation [41]. We used induced VSMCs with PDGF-BB to simulate the pathophysiologic environment of VSMCs in the vessel wall during IA. Metformin prevented de-differentiation of VSMCs by increasing AMPK phosphorylation while simultaneously inhibiting their proliferation, migration, and apoptosis.

Another interesting finding of the present work is that ACC was activated by AMPK and blocked the phenotypic switching of VSMCs. ACC is an enzyme that acts downstream of AMPK in the regulation of fatty acid metabolism [19]. It was recently reported that AMPK and ACC regulate endothelial cell differentiation and migration [20]. In our study, VSMCs were treated with the small molecular inhibitor ND-646, which is known to suppress ACC phosphorylation [28]; this resulted in the downregulation of α-SMA and SM22α and stimulated the production of IL-1β, IL-6, MMP-3, MMP-9, iNOS, and TNF-α, effects that could not be reversed by metformin. Thus, the AMPK/ACC axis mediates phenotype modulation of VSMCs in IAs.

## Conclusion

**Fig. 8:**
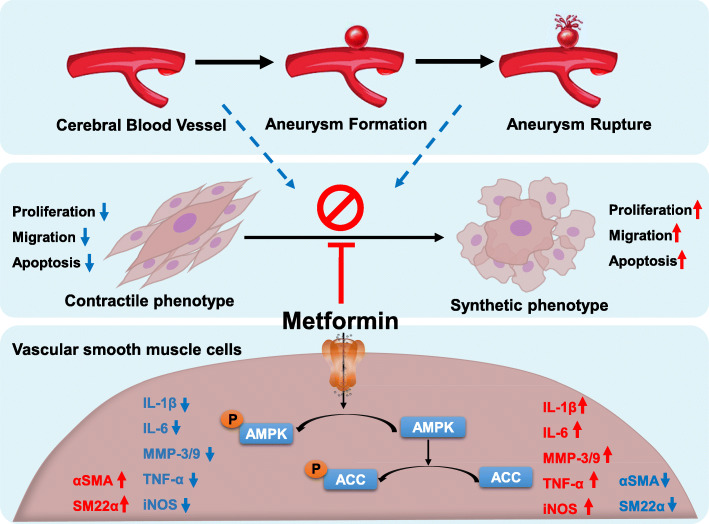
Schematic illustrating how metformin modulates the phenotype switching in vascular smooth muscle cell (VSMC) to inhibit the pathogenesis of intracranial aneurysm. Metformin could inhibit the phenotype switching of VSMC from contractile to synthetic phenotype by activating AMPK/ACC pathway, resulting in upregulation of VSMC-specific markers αSMA, SM22α, and decreased expression of pro-inflammatory cytokines IL-1β, IL-6, MMP-3/9, TNF-α, and iNOS, suppressing formation and progression of intracranial aneurysm ultimately

In summary, we demonstrated that metformin decreased the incidence and rupture rate of IAs in a rat model by activating AMPK/ACC signaling and inducing phenotype switching of VSMCs in the IA vessel wall, which was corroborated by observations in human IA and STA samples (Fig 8). These findings highlight the clinical potential of metformin for preventing cerebral aneurysm formation and aneurysmal rupture.

## Data Availability

Data generated and analyzed as part of this study are included in the manuscript or are available upon request from the corresponding author.

## Abbreviations

AMPK: 5′ AMP-activated protein kinase
ACC: Acetyl-CoA carboxylase
VSMC: Vascular smooth muscle cell
IA: Intracranial aneurysm
PDGF: Platelet-derived growth factor
STA: Superficial temporal artery
α-SMA: α-Smooth muscle actin
SM-22α: Smooth muscle protein 22α
MMP: Matrix metalloproteinase
IL: Interleukin
